# Treatment Optimization with Add-On Kampo Therapy in Highly Refractory Migraine Patients with Inadequate Response or Re-Worsening During CGRP Monoclonal Antibody Therapy: A Real-World Treatment Episode Analysis

**DOI:** 10.3390/jcm15218633

**Published:** 2026-11-05

**Authors:** Takafumi Tanei, Satoshi Yamashita, Satoshi Maesawa, Tomotaka Ishizaki, Takahiro Suzuki, Shun Yamamoto, Satoshi Shinoda, Toshihiko Wakabayashi, Ryuta Saito

**Affiliations:** 1 Department of Neurosurgery, Nagoya University Graduate School of Medicine, Nagoya 466-8560, Aichi, Japan; 2 Specialized Headache Clinic, Nagoya Garden Clinic, Nagoya 451-0051, Aichi, Japan; 3 Department of Neurosurgery, National Hospital Organization, Nagoya Medical Center, Nagoya 460-0001, Aichi, Japan

**Keywords:** migraine, calcitonin gene-related peptide monoclonal antibodies, Kampo medicine, herbal medicine, refractory migraine, re-worsening, medication-overuse headache

## Abstract

**Background/Objectives:** Although calcitonin gene-related peptide monoclonal antibodies (CGRP-mAbs) have improved migraine prevention, some patients continue to experience inadequate response or re-worsening during treatment. This study evaluated the effectiveness of add-on Kampo therapy in migraine patients receiving CGRP-mAb treatment. **Methods:** This retrospective observational study included 39 migraine patients who received add-on Kampo therapy during ongoing CGRP-mAb treatment, contributing 55 treatment episodes. The primary endpoint was the change in monthly headache days (MHD) from baseline to 1 month after Kampo initiation. Secondary outcomes included migraine frequency, headache-related disability, quality of life, acute medication use, and work productivity. A responder was defined as achieving a ≥30% reduction in MHD at 1 month. **Results:** The study population was highly refractory, with chronic migraine present in 79.5% of patients and medication-overuse headache in 56.4%. Among treatment episodes, 72.7% experienced re-worsening after an initial response to CGRP-mAb treatment. Median MHD was 14.0 days after CGRP-mAb treatment and subsequently increased to 16.0 days before Kampo initiation. After Kampo initiation, median MHD decreased to 10.0 days at 1 month. Significant improvements were also observed at 1 month in headache-related disability, quality of life, acute medication use, and presenteeism. The ≥30% and ≥50% responder rates at 1 month were 41.8% and 12.7%, respectively. Re-worsening after an initial CGRP-mAb response showed an exploratory association with treatment response in the Firth penalized logistic regression model. **Conclusions:** Add-on Kampo therapy was associated with improvements in headache burden, quality of life, acute medication use, and work productivity in patients with inadequate response or re-worsening during CGRP-mAb treatment. These hypothesis-generating findings suggest a potential role for add-on Kampo therapy as a treatment optimization strategy and warrant confirmation in prospective controlled studies.

## 1. Introduction

Migraine is one of the most prevalent and disabling neurological disorders worldwide and is associated with substantial impairment in quality of life, social functioning, and work productivity. It remains a leading cause of disability, particularly among young and middle-aged adults [1,2]. The introduction of calcitonin gene-related peptide monoclonal antibodies (CGRP-mAbs) has substantially improved preventive treatment for migraine. Clinical trials and real-world studies have demonstrated favorable efficacy and tolerability of CGRP-mAbs in both episodic and chronic migraine (CM) [3,4,5]. Real-world evidence has further confirmed their effectiveness in difficult-to-treat populations, including patients with medication-overuse headache (MOH) [6].

Despite these advances, a subset of patients continue to experience inadequate response or re-worsening after an initial response during ongoing CGRP-mAb treatment. In real-world practice, maintaining long-term treatment persistence and sustained efficacy remains challenging [7]. Patients with CM, MOH, a history of multiple preventive medication use, and inadequate response to CGRP-targeted therapies represent a particularly difficult-to-treat population [6,8,9]. Therapeutic options become increasingly limited when patients remain symptomatic despite CGRP-mAb treatment or experience re-worsening after an initial response. Therefore, additional treatment strategies are needed for patients with persistent migraine burden despite ongoing CGRP-mAb therapy [8,9].

In Japan, Kampo medicine, a traditional Japanese herbal medicine, is sometimes used in routine clinical practice as an adjunctive treatment when conventional pharmacological therapies do not provide adequate symptom control. Kampo formulations are prescribed for diverse clinical conditions, with headache disorders and neuropathic pain representing examples relevant to the present study [10]. In addition, pharmacological studies have demonstrated that Kampo-derived herbal compounds interact with multiple neurotransmitter systems, including GABA_A_ and 5-HT_3A_ receptors [11]. Several Kampo formulations have shown potential benefits in patients with migraine and tension-type headache and are occasionally prescribed for patients with persistent symptoms despite standard preventive therapies [12,13,14].

The mechanisms by which Kampo formulations may provide additional benefit in patients with refractory migraine remain incompletely understood. However, experimental evidence suggests that certain Kampo medicines, including Yokukansan, may influence pain processing through modulation of GABAergic and serotonergic neurotransmission, regulation of neuronal excitability, and attenuation of central sensitization [10,11]. These mechanisms may differ from CGRP pathway inhibition and could therefore provide complementary therapeutic effects in some patients who remain symptomatic despite CGRP-mAb treatment.

However, evidence regarding the effectiveness of add-on Kampo therapy in patients with inadequate response or re-worsening during ongoing CGRP-mAb treatment remains limited. Furthermore, its clinical utility has not been adequately investigated in highly refractory migraine populations characterized by CM, MOH, a history of multiple preventive medication use, and prior CGRP-mAb switching.

Therefore, this study aimed to evaluate the effectiveness of add-on Kampo therapy in highly refractory migraine patients with inadequate response or re-worsening during CGRP-mAb treatment. We assessed changes in MHD, monthly migraine days (MMD), headache-related disability, quality of life, acute medication use (AMU), and work productivity following the initiation of Kampo therapy.

## 2. Materials and Methods

### 2.1. Study Design and Setting

**Figure 1:**
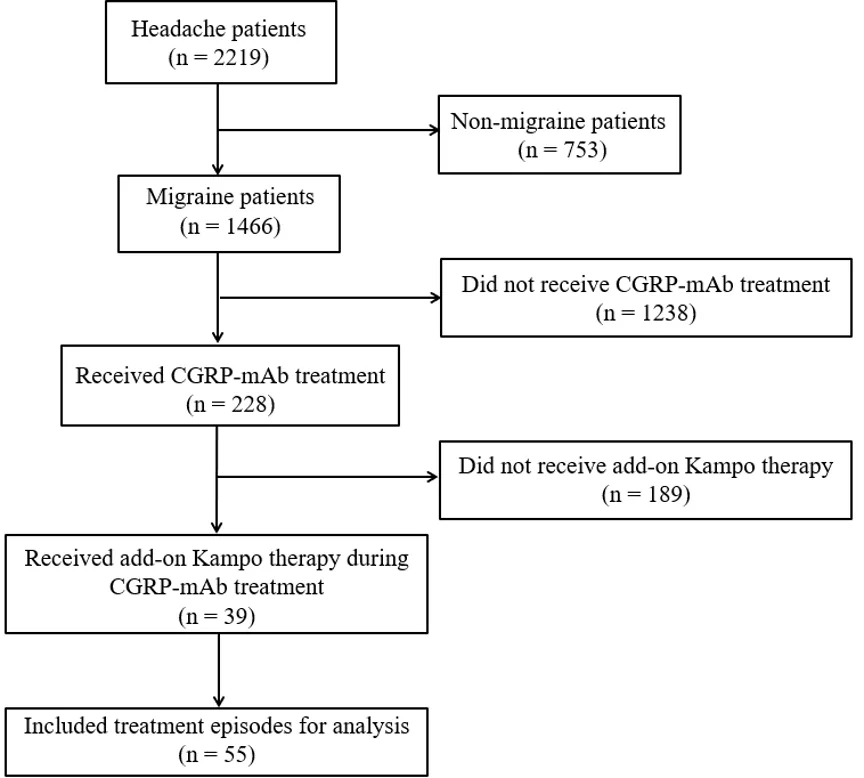
Study flowchart and patient selection process.

This retrospective observational study evaluated the effectiveness of add-on Kampo therapy in migraine patients with inadequate response or re-worsening during CGRP-mAb treatment. The study was conducted at a specialized headache outpatient clinic. Among 2219 headache patients treated between January 2022 and August 2026, 1466 were diagnosed with migraine according to the International Classification of Headache Disorders, 3rd edition (ICHD-3) [15]. Of these, 228 received CGRP-mAb treatment. Add-on Kampo therapy was prescribed to 39 patients who experienced inadequate response or re-worsening during CGRP-mAb treatment, resulting in 55 treatment episodes included in the analysis (Figure 1). The unit of analysis was the treatment episode. Patients could contribute multiple episodes if different Kampo formulations were prescribed sequentially during the observation period. During the primary 1-month evaluation period after Kampo initiation, the CGRP-mAb agent and dosing schedule, other preventive medications, and MOH management remained unchanged. The study was approved by the Ethics Review Committee of Nagoya University Graduate School of Medicine (approval number: 2024-0251). The requirement for written informed consent was waived because of the retrospective study design.

Flowchart illustrating patient selection for the study. Among 2219 headache patients, 1466 were diagnosed with migraine and 228 received CGRP monoclonal antibody (CGRP-mAb) therapy. Thirty-nine patients received add-on Kampo therapy during ongoing CGRP-mAb treatment, contributing 55 treatment episodes that were included in the final analysis.

### 2.2. Kampo Therapy

**Figure 2:**
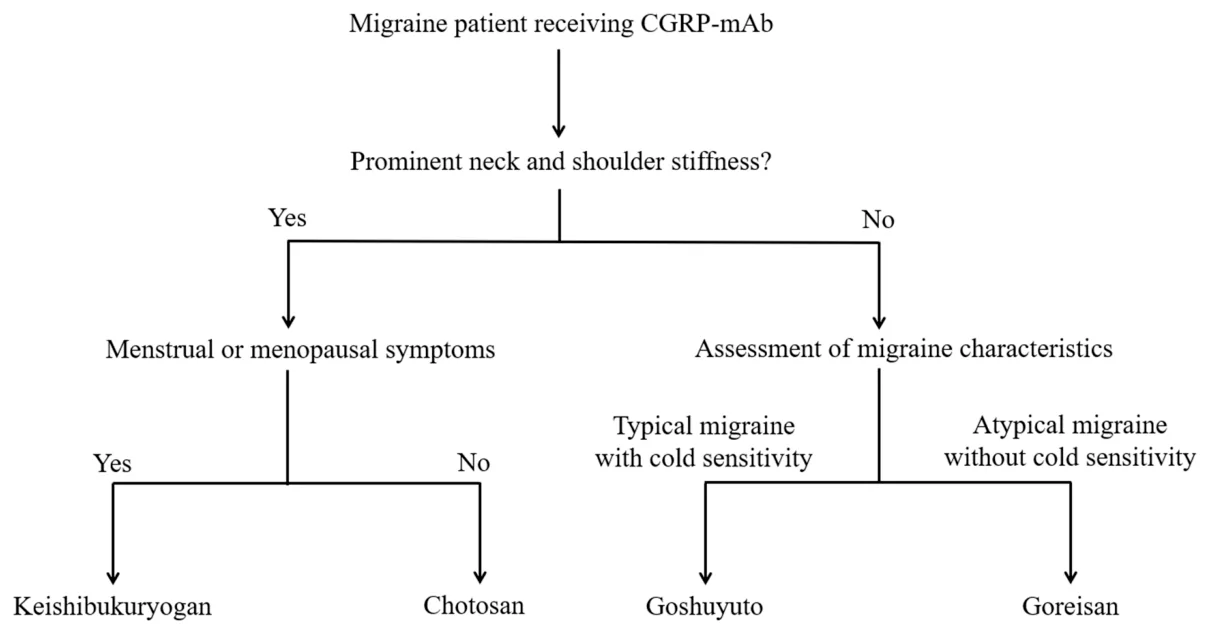
Symptom-based algorithm for the selection of Kampo formulations in migraine patients receiving CGRP-mAb therapy.

Kampo formulations were selected according to a predefined symptom-based algorithm previously reported (Figure 2) [14]. Chotosan, Goreisan, Goshuyuto, and Keishibukuryogan are multi-herbal formulations with different traditional indications and pharmacological properties; in the present study, they were selected according to individual migraine characteristics and accompanying symptoms.

Kampo formulations were selected as add-on therapy according to migraine characteristics and accompanying symptoms, including cold sensitivity, neck and shoulder stiffness, and menstrual or menopausal symptoms.

### 2.3. Data Collection

The following demographic and clinical variables were collected from medical records. Migraine diagnoses were established by physicians at the specialized headache clinic according to the ICHD-3 criteria [15]. Psychiatric comorbidity was defined as a documented clinical diagnosis of anxiety disorder and/or depressive disorder in the medical records. Patient characteristics included age, sex, family history of migraine, presence of migraine with aura, and psychiatric comorbidities. Migraine was classified as episodic migraine (EM), high-frequency episodic migraine (HFEM), or CM based on the diagnosis and monthly headache frequency documented at the initial clinical presentation. MOH was defined according to ICHD-3 criteria [15]. For episode-based analyses, patient-level CM and MOH status were assigned to each corresponding treatment episode. Treatment episode characteristics included a history of multiple preventive medication use, CGRP-mAb switch history, time from initiation of CGRP-mAb therapy to Kampo initiation, reason for Kampo initiation (primary inadequate response or re-worsening after an initial response), and the prescribed Kampo formulation. Clinical outcome data were obtained from headache diaries and patient-reported outcome assessments.

### 2.4. Outcome Measures

#### 2.4.1. Primary Outcome

The primary outcome was the change in MHD from baseline (immediately before Kampo initiation) to 1 month after Kampo initiation.

#### 2.4.2. Secondary Outcomes

Secondary outcomes included MMD, Headache Impact Test-6 (HIT-6) [16], Migraine-Specific Quality of Life Questionnaire (MSQ) [17], total AMU days, triptan use days, presenteeism score, and absenteeism score [18]. For both the HIT-6 and MSQ, higher scores indicated greater migraine-related impairment; therefore, decreases in these scores indicated improvement. The untransformed sum of the 14 MSQ item scores was used. These outcomes were assessed before CGRP-mAb treatment, at 1 month after CGRP-mAb initiation, immediately before Kampo initiation (baseline), and at 1, 2, and 3 months after Kampo initiation.

### 2.5. Definition of Headache Day and Migraine Day

A headache day was defined as any calendar day with headache recorded in the headache diary [18]. A migraine day was defined as any calendar day on which the recorded headache fulfilled the ICHD-3 diagnostic criteria for migraine [15].

### 2.6. Presenteeism and Absenteeism Scores

Presenteeism and absenteeism were assessed using headache diary-derived scores previously developed in clinical practice [18]. The absenteeism score was calculated as (full-day absence × 1.0) + (half-day absence × 0.5). The presenteeism score was calculated as (major productivity loss × 1.0) + (minor productivity loss × 0.5). Higher scores indicated greater impairment in work productivity.

### 2.7. Definition of Responder

A responder was defined as a treatment episode achieving a ≥30% reduction in MHD at 1 month after Kampo initiation. A 30% threshold was selected because the study population consisted of highly refractory migraine patients with substantial disease burden and a history of multiple preventive medication use.

### 2.8. Statistical Analysis

Continuous variables are presented as medians (interquartile ranges [IQRs]) or means ± standard deviations (SDs), as appropriate. Comparisons between paired observations were performed using the Wilcoxon signed-rank test. Changes in clinical outcomes were evaluated for CGRP pre-treatment versus CGRP post-treatment and for baseline versus 1, 2, and 3 months after Kampo initiation. For analyses of change from baseline, 95% confidence intervals (CIs) were estimated using bootstrap resampling with 10,000 iterations.

Responder and non-responder treatment episodes were compared using the Mann–Whitney U test for continuous variables and Fisher’s exact test for categorical variables. To explore factors associated with treatment response, Firth penalized multivariable logistic regression analysis was performed using responder status (≥30% reduction in MHD at 1 month) as the dependent variable. This approach was selected to reduce small-sample bias and address potential separation arising from the limited number of responders and the highly unbalanced distribution of re-worsening among responders. The primary penalized model included MOH status, CGRP-mAb switch history, re-worsening after an initial response, and time from CGRP-mAb initiation to Kampo initiation as explanatory variables. Penalized odds ratios (ORs), 95% CIs, and *p* values were estimated using the Firth penalized likelihood approach. In addition, a reduced Firth penalized logistic regression model including only re-worsening and MOH status was performed as a supplementary analysis to assess the robustness of the findings with fewer predictors.

To account for multiple comparisons in longitudinal outcome analyses, Bonferroni correction was applied. Unadjusted *p* values are presented, and comparisons that remained statistically significant after Bonferroni correction are indicated by dagger symbols (†). Missing values were not imputed, and available-case analyses were performed for each outcome measure. All statistical tests were two-sided.

All statistical analyses were conducted using EZR (version 1.68; Saitama Medical Center, Jichi Medical University, Saitama, Japan), a graphical user interface for R (version 4.3.1; The R Foundation for Statistical Computing, Vienna, Austria), which is a modified version of R Commander (version 2.9-1) designed to add statistical functions frequently used in biostatistics [19]. Firth penalized logistic regression analyses were performed using the logistf package (version 1.26.1) in R through the EZR interface. 

As a sensitivity analysis, multiple linear regression analysis was performed using the percentage reduction in MHD at 1 month after Kampo initiation as a continuous outcome variable. The same explanatory variables used in the multivariable logistic regression analysis were included in the multiple linear regression model. This study is reported in accordance with the STROBE statement for observational studies.

## 3. Results

### 3.1. Patient and Treatment Episode Characteristics

A total of 39 patients contributed 55 treatment episodes and were included in the final analysis (Figure 1). Baseline patient and treatment episode characteristics are summarized in Table 1. The mean age was 43.8 ± 12.2 years, and 33 patients (84.6%) were female. CM was present in 31 patients (79.5%), and MOH was observed in 22 patients (56.4%). Among the 55 treatment episodes, 42 (76.4%) had a history of multiple preventive medication use, and 31 (56.4%) had undergone prior CGRP-mAb switching. The median duration from CGRP-mAb initiation to Kampo initiation was 10.0 months (IQR 5.0–16.0). The most common reason for Kampo initiation was re-worsening after an initial response to CGRP-mAb treatment (40 episodes, 72.7%), whereas primary inadequate response accounted for 15 episodes (27.3%). Chotosan was the most frequently prescribed Kampo formulation (23 episodes, 41.8%), followed by Goreisan (13 episodes, 23.6%), Goshuyuto (9 episodes, 16.4%), Keishibukuryogan (5 episodes, 9.1%), and other Kampo formulations (5 episodes, 9.1%). No changes in the CGRP-mAb agent or dosing schedule, other preventive medications, or MOH management occurred during the primary 1-month evaluation period.

**Table 1:** Baseline characteristics of patients and treatment episodes.

**A. Patient Characteristics**	***n*** ** = 39**
Age (years), mean ± SD	43.8 ± 12.2
Female sex, *n* (%)	33 (84.6%)
Family history of migraine, *n* (%)	23 (59.0%)
Migraine with aura, *n* (%)	21 (53.8%)
Psychiatric comorbidity, *n* (%)	5 (12.8%)
Migraine classification	
Episodic migraine, *n* (%)	3 (7.7%)
High-frequency episodic migraine, *n* (%)	5 (12.8%)
Chronic migraine, *n* (%)	31 (79.5%)
Medication-overuse headache, *n* (%)	22 (56.4%)
**B. Treatment Episode Characteristics**	***n*** ** = 55**
Multiple preventive medication history, *n* (%)	42 (76.4%)
CGRP-mAb switch history, *n* (%)	31 (56.4%)
Time from CGRP-mAb initiation to Kampo initiation, months	10.0 (5.0, 16.0)
Re-worsening after an initial response, *n* (%)	40 (72.7%)
Primary inadequate response, *n* (%)	15 (27.3%)
Kampo formulations, *n* (%)	
Chotosan	23 (41.8%)
Goreisan	13 (23.6%)
Goshuyuto	9 (16.4%)
Keishibukuryogan	5 (9.1%)
Other formulations	5 (9.1%)

Values are presented as mean ± standard deviation (SD), median (IQR), or *n* (%). Patient characteristics were summarized for 39 patients, whereas treatment-related characteristics were summarized for 55 treatment episodes. Psychiatric comorbidity included anxiety disorder and/or depressive disorder. CGRP-mAb: calcitonin gene-related peptide monoclonal antibody.

### 3.2. Changes in Clinical Outcomes

#### 3.2.1. Monthly Headache Days

**Figure 3:**
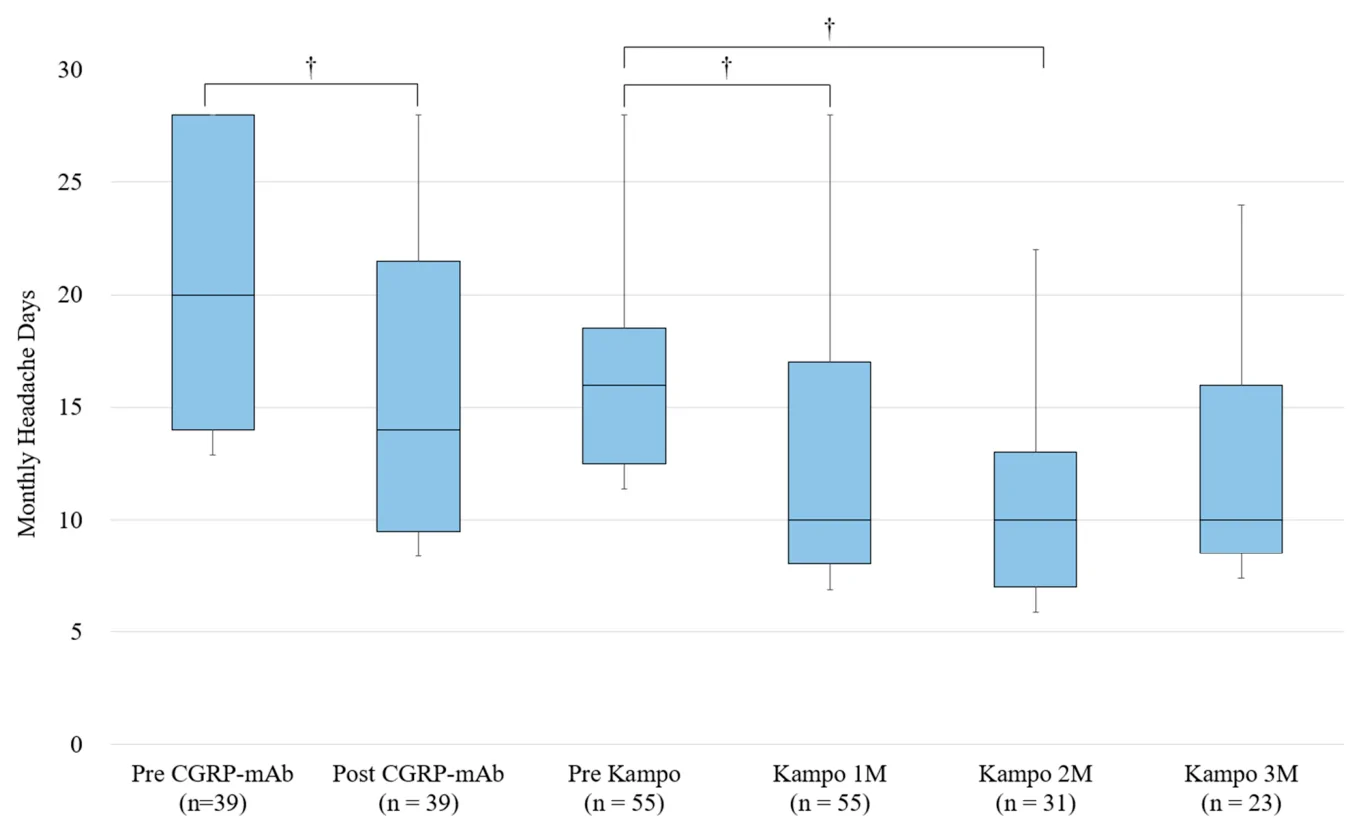
Longitudinal changes in monthly headache days during CGRP-mAb and add-on Kampo therapy. Monthly headache days before and after CGRP monoclonal antibody (CGRP-mAb) treatment, immediately before Kampo initiation, and at 1, 2, and 3 months after Kampo initiation. Data are presented as medians with interquartile ranges. † *p* < 0.001 for the comparisons indicated by brackets.

MHD, the primary outcome of the study, decreased after Kampo initiation (Figure 3). Median MHD decreased from 20.0 (IQR 14.0–28.0) days before CGRP-mAb treatment to 14.0 (IQR 9.5–21.5) days after CGRP-mAb treatment. However, MHD subsequently increased to 16.0 (IQR 12.5–18.5) days before Kampo initiation, reflecting inadequate response or re-worsening during ongoing CGRP-mAb treatment. After Kampo initiation, median MHD decreased to 10.0 (IQR 8.0–17.0) days at 1 month, 10.0 (IQR 7.0–13.0) days at 2 months, and 10.0 (IQR 8.0–16.0) days at 3 months. In the longitudinal time-point comparisons, the reductions remained statistically significant after Bonferroni correction at 1 and 2 months, but not at 3 months. Changes from baseline are summarized separately. Median changes in MHD were −3.0 (IQR −6.0–−1.0) days at 1 month, −4.0 (IQR −6.0–−3.0) days at 2 months, and −4.0 (IQR −6.0–1.0) days at 3 months; the reductions from baseline remained statistically significant after Bonferroni correction at all three follow-up time points.

#### 3.2.2. Secondary Outcomes

Longitudinal changes in secondary outcomes are summarized in Table 2. Median MMD decreased from 4.0 (IQR 2.0–7.8) days before CGRP-mAb treatment to 2.0 (IQR 0.3–4.0) days after treatment. Before Kampo initiation, median MMD was 1.0 (IQR 0.0–5.0) days and remained low throughout follow-up, with values of 1.0 (IQR 0.0–3.0), 0.0 (IQR 0.0–1.5), and 1.0 (IQR 0.0–3.0) days at 1, 2, and 3 months, respectively.

Headache-related disability and migraine-specific quality of life improved after Kampo initiation. Median HIT-6 scores decreased from 59.0 (IQR 52.0–61.0) at baseline to 54.0 (IQR 48.0–59.0) at 1 month, 54.0 (IQR 48.0–58.5) at 2 months, and 57.0 (IQR 52.0–60.0) at 3 months. Median changes from baseline ranged from −2.0 to −3.0 points (Table 3), and a statistically significant improvement after Bonferroni correction was observed at 1 month.

Similarly, median MSQ scores decreased from 25.0 (IQR 20.0–33.0) at baseline to 21.0 (IQR 18.0–26.0) at 1 month, 20.5 (IQR 19.0–24.3) at 2 months, and 22.0 (IQR 19.0–25.0) at 3 months. Median changes from baseline ranged from −1.5 to −4.0 points (Table 3), with a statistically significant improvement after Bonferroni correction at 1 month.

AMU also decreased following Kampo therapy. Median total AMU decreased from 12.0 (IQR 8.0–18.0) days at baseline to 8.0 (IQR 6.0–14.8) days at 1 month, 9.5 (IQR 6.3–12.8) days at 2 months, and 10.0 (IQR 6.0–13.5) days at 3 months. Median changes from baseline ranged from −3.0 to −5.0 days (Table 3), and these improvements remained statistically significant after Bonferroni correction throughout follow-up. Median AMU triptan also decreased and remained significantly lower at 1, 2, and 3 months after Bonferroni correction.

Work productivity impairment, assessed using the presenteeism score, improved after treatment, with a statistically significant improvement after Bonferroni correction at 1 month. However, this effect was not maintained at later time points. In contrast, absenteeism scores remained largely unchanged throughout follow-up and did not show statistically significant improvement (Table 2 and Table 3).

**Table 2:** Longitudinal changes in secondary outcomes during CGRP-mAb and add-on Kampo therapy.

	CGRP Pre		CGRP Post 1M		Pre vs. Post	Kampo Pre		Kampo Post 1M		Pre vs. 1M	Kampo Post 2M		Pre vs. 2M	Kampo Post 3M		Pre vs. 3M
	Median (IQR)	*n*	Median (IQR)	*n*	*p* Value	Median (IQR)	*n*	Median (IQR)	*n*	*p* Value	Median (IQR)	*n*	*p* Value	Median (IQR)	*n*	*p* Value
MMD	4.0 (2.0, 7.8)	39	2.0 (0.3, 4.0)	39	<0.001 ^†^	1.0 (0.0, 5.0)	49	1.0 (0.0, 3.0)	49	0.088	0.0 (0.0, 1.5)	31	0.225	1.0 (0.0, 3.0)	23	0.816
HIT-6	64.0 (60.0, 66.0)	38	56.5 (50.0, 62.0)	38	<0.001 ^†^	59.0 (52.0, 61.0)	53	54.0 (48.0, 59.0)	49	<0.001 ^†^	54.0 (48.0, 58.5)	28	0.007	57.0 (52.0, 60.0)	24	0.296
MSQ	36.0 (28.0, 44.5)	39	23.0 (18.5, 28.5)	39	<0.001 ^†^	25.0 (20.0, 33.0)	53	21.0 (18.0, 26.0)	49	<0.001 ^†^	20.5 (19.0, 24.3)	28	0.016	22.0 (19.0, 25.0)	23	0.019
AMU Total	17.0 (9.3, 28.0)	38	9.0 (5.0, 19.5)	39	<0.001 ^†^	12.0 (8.0, 18.0)	50	8.0 (6.0, 14.8)	50	<0.001 ^†^	9.5 (6.3, 12.8)	30	<0.001 ^†^	10.0 (6.0, 13.5)	23	<0.001 ^†^
AMU Triptan	7.0 (3.0, 11.0)	39	4.0 (2.0, 7.5)	39	0.003	7.0 (4.0, 11.0)	50	5.0 (2.0, 7.0)	50	<0.001 ^†^	6.0 (3.3, 8.0)	30	<0.001 ^†^	6.0 (2.3, 8.0)	23	<0.001 ^†^
Presenteeism score	3.5 (2.0, 8.0)	23	2.0 (1.0, 4.0)	29	0.009	5.3 (1.8, 7.6)	44	3.0 (1.0, 5.3)	44	<0.001 ^†^	2.5 (1.5, 6.0)	29	0.009	2.8 (0.6, 5.3)	23	0.008
Absenteeism score	0.0 (0.0, 0.6)	24	0.0 (0.0, 0.0)	30	0.407	0.0 (0.0, 0.5)	44	0.0 (0.0, 0.0)	44	0.033	0.0 (0.0, 0.0)	29	0.437	0.0 (0.0, 0.0)	22	1.000

Sample sizes shown at each time point represent available observations. Wilcoxon signed-rank tests were performed using paired observations only. Comparisons were performed between CGRP pre and CGRP post, and between Kampo pre and each post-treatment time point. † Remained statistically significant after Bonferroni correction. MMD: monthly migraine days, HIT-6: Headache Impact Test-6, MSQ: Migraine-Specific Quality of Life Questionnaire, AMU: Acute Medication Use, M: month.

**Table 3:** Changes in clinical outcomes during CGRP-mAb and add-on Kampo therapy.

Outcome	Change	Median Change (IQR)	Paired *n*	95% CI	*p* Value
ΔMHD	CGRP post 1M–CGRP pre	−4.0 (−7.5, −2.0)	39	[−6.0, −3.0]	<0.001 ^†^
	1M–Kampo pre	−3.0 (−6.0, −1.0)	55	[−5.0, −2.0]	<0.001 ^†^
	2M–Kampo pre	−4.0 (−6.0, −3.0)	31	[−6.0, −4.0]	<0.001 ^†^
	3M–Kampo pre	−4.0 (−6.0, 1.0)	23	[−6.0, −1.0]	<0.001 ^†^
ΔMMD	CGRP post 1M–CGRP pre	−2.0 (−4.0, 0.0)	38	[−3.0, −1.0]	<0.001 ^†^
	1M–Kampo pre	0.0 (−1.0, 1.0)	49	[−1.0, 0.0]	0.219
	2M–Kampo pre	0.0 (−1.0, 0.0)	31	[−1.0, 0.0]	0.246
	3M–Kampo pre	0.0 (−1.0, 1.0)	23	[0.0, 1.0]	0.628
ΔHIT-6	CGRP post 1M–CGRP pre	−7.5 (−12.0, −2.3)	38	[−12.0, −4.0]	<0.001 ^†^
	Kampo post 1M–Kampo pre	−2.0 (−8.0, 1.0)	49	[−6.0, 0.0]	<0.001 ^†^
	Kampo post 2M–Kampo pre	−3.0 (−8.0, 1.0)	28	[−7.0, 0.0]	0.005
	Kampo post 3M–Kampo pre	−2.0 (−4.5, 2.5)	23	[−4.0, 1.0]	0.230
ΔMSQ	CGRP post 1M–CGRP pre	−8.0 (−17.0, −3.5)	39	[−14.0, −6.0]	<0.001 ^†^
	Kampo post 1M–Kampo pre	−3.0 (−7.0, 0.0)	49	[−5.0, −1.0]	<0.001 ^†^
	Kampo post 2M–Kampo pre	−1.5 (−7.5, 0.3)	28	[−5.0, 0.0]	0.010
	Kampo post 3M–Kampo pre	−4.0 (−7.0, 1.0)	23	[−7.0, 0.0]	0.012
ΔAMU total	CGRP post 1M–CGRP pre	−5.5 (−12.0, −3.0)	38	[−8.5, −4.0]	<0.001 ^†^
	Kampo post 1M–Kampo pre	−3.0 (−6.0, −1.0)	50	[−4.0, −2.0]	<0.001 ^†^
	Kampo post 2M–Kampo pre	−5.0 (−6.0, 0.8)	30	[−6.0, −1.0]	0.003
	Kampo post 3M–Kampo pre	−4.0 (−6.5, −3.0)	23	[−6.0, −3.0]	<0.001 ^†^
ΔPresenteeism score	CGRP post 1M–CGRP pre	−2.5 (−3.5, −0.3)	23	[−3.5, −0.5]	0.005
	Kampo post 1M–Kampo pre	−1.3 (−3.0, 0.5)	44	[−2.5, 0.0]	<0.001 ^†^
	Kampo post 2M–Kampo pre	−1.0 (−3.0, 0.0)	29	[−2.0, 0.0]	0.005
	Kampo post 3M–Kampo pre	−1.0 (−2.5, 0.4)	23	[−2.5, 0.0]	0.007
ΔAbsenteeism score	CGRP post 1M–CGRP pre	0.0 (0.0, 0.0)	24	[0.0, 0.0]	0.604
	Kampo post 1M–Kampo pre	0.0 (0.0, 0.0)	44	[0.0, 0.0]	0.060
	Kampo post 2M–Kampo pre	0.0 (0.0, 0.0)	29	[0.0, 0.0]	0.522
	Kampo post 3M–Kampo pre	0.0 (0.0, 0.0)	22	[0.0, 0.0]	0.665

Values represent changes between the indicated time points. 95% confidence intervals (CIs) were estimated using bootstrap resampling (10,000 iterations), and p values were calculated using the Wilcoxon signed-rank test. † Remained statistically significant after Bonferroni correction. CGRP-mAb: calcitonin gene-related peptide monoclonal antibody, IQR: interquartile range, MHD: monthly headache days, MMD: monthly migraine days, HIT-6: Headache Impact Test-6, MSQ: Migraine-Specific Quality of Life Questionnaire, AMU: Acute Medication Use, M: month.

### 3.3. Responder Analysis

Responder rates based on reductions in MHD are summarized in Table 4. At 1 month after Kampo initiation, the ≥30% responder rate was 41.8% (23/55; 95% CI, 28.7–55.9%), whereas the ≥50% responder rate was 12.7% (7/55; 95% CI, 5.3–24.5%). At 2 months, the corresponding ≥30% and ≥50% responder rates were 54.8% (17/31; 95% CI, 36.0–72.7%) and 22.6% (7/31; 95% CI, 9.6–41.1%), respectively. At 3 months, the corresponding ≥30% and ≥50% responder rates were 39.1% (9/23; 95% CI, 19.7–61.5%) and 8.7% (2/23; 95% CI, 1.1–28.0%), respectively.

### 3.4. Factors Associated with Treatment Response

Clinical characteristics of responder and non-responder treatment episodes are summarized in Table 5. Responders more frequently experienced re-worsening after an initial response to CGRP-mAb therapy (95.7% vs. 56.3%, *p* = 0.002), whereas patient-level MOH status was less common among responders (43.5% vs. 78.1%, *p* = 0.012). CM was more prevalent among non-responders than responders (93.8% vs. 73.9%), although this difference did not reach statistical significance (*p* = 0.057). No significant differences were observed with respect to age, CGRP-mAb switch history, or time from CGRP-mAb initiation to Kampo initiation.

### 3.5. Multivariable Analysis of Factors Associated with Treatment Response

In the four-variable Firth penalized logistic regression model, re-worsening after an initial response to CGRP-mAb therapy showed an exploratory association with treatment response (penalized OR 14.581, 95% CI 2.15–180.80, *p* = 0.005) (Table 6). MOH status, CGRP-mAb switch history, and time from CGRP-mAb initiation to Kampo initiation were not significantly associated with treatment response. In the reduced two-variable Firth penalized logistic regression model including re-worsening and MOH status, re-worsening remained associated with treatment response (penalized OR 8.179, 95% CI 1.63–81.74, *p* = 0.009), whereas MOH status was not significantly associated with treatment response (penalized OR 0.388, 95% CI 0.11–1.30, *p* = 0.125) (Supplementary Table S1).

In a sensitivity analysis using the percentage reduction in MHD at 1 month as a continuous outcome, re-worsening after an initial response to CGRP-mAb therapy was positively associated with a greater percentage reduction in MHD (β = 0.190, *p* = 0.015), whereas MOH status was associated with a smaller percentage reduction in MHD (β = −0.123, *p* = 0.042) (Table 7).

### 3.6. Adverse Events

No adverse events related to Kampo therapy were documented among the 55 treatment episodes included in this study.

## 4. Discussion

In this retrospective treatment episode-based study, we evaluated the effectiveness of add-on Kampo therapy in highly refractory migraine patients who experienced either inadequate response or re-worsening during ongoing CGRP-mAb treatment. The principal findings were as follows. First, MHD, the primary outcome, improved after initiation of Kampo therapy. Second, headache-related disability and migraine-specific quality of life improved. Third, total AMU days decreased after Kampo initiation. Finally, work productivity impairment improved during follow-up. Collectively, these findings suggest a potential role for add-on Kampo therapy as a treatment optimization strategy for highly refractory migraine patients who remain symptomatic despite CGRP-mAb treatment.

The introduction of CGRP-mAb therapy has substantially improved preventive treatment for migraine and expanded therapeutic options for patients with difficult-to-treat migraine. Nevertheless, a subset of patients continue to experience inadequate response or re-worsening after an initial response during long-term treatment, and treatment of these patients often remains challenging because several preventive treatment options have already been tried [3,4,5,7,8,9]. The present cohort represented a highly refractory migraine population. Previous consensus statements have recognized refractory migraine as a major unmet clinical challenge [9,20]. Among the 39 patients included in this study, 79.5% had CM and 56.4% had MOH. In addition, 76.4% of treatment episodes had a history of multiple preventive medication use, 56.4% had undergone prior CGRP-mAb switching, and 72.7% were initiated because of re-worsening after an initial response to CGRP-mAb treatment. These findings indicate that most patients had already exhausted several established preventive treatment options before Kampo therapy was considered. Previous real-world studies have shown that maintenance of long-term efficacy remains challenging during CGRP-targeted therapy and that a substantial proportion of patients remain symptomatic despite switching between CGRP-targeted agents [7,8,9,21]. In the present study, the median duration from CGRP-mAb initiation to Kampo initiation was 10.0 months, suggesting that Kampo therapy was generally introduced after a prolonged period of clinical observation rather than during the early treatment phase.

In the present study, add-on Kampo therapy was associated with improvements in several clinically relevant outcomes, including MHD, HIT-6, MSQ, total AMU days, and presenteeism score. Importantly, these improvements were observed despite the highly refractory nature of the study population. Among the evaluated outcomes, reduction in MHD is particularly important because headache frequency remains one of the most widely accepted indicators of treatment effectiveness in migraine prevention studies [3,4,5]. In the present cohort, headache frequency initially improved after CGRP-mAb treatment but subsequently worsened before Kampo initiation, suggesting inadequate response or re-worsening during ongoing therapy. Following Kampo initiation, improvements were observed not only in headache frequency but also in headache-related disability, quality of life, and acute medication use, suggesting a potential additional benefit beyond that achieved with CGRP-mAb treatment alone. Previous migraine studies have emphasized that treatment success should be assessed not only by headache frequency but also by patient-reported outcomes and functional measures [16,17,22]. Although statistically significant improvements in HIT-6 and MSQ were observed at 1 month, the absolute magnitude of these changes was modest and should be interpreted cautiously. Total AMU decreased from 12.0 days at baseline to 8.0 days at 1 month, indicating a reduction in rescue medication use, but subsequently increased numerically to 9.5 days at 2 months and 10.0 days at 3 months. This pattern should be interpreted cautiously because the number of available observations decreased during follow-up and does not necessarily indicate within-patient re-worsening. Nevertheless, this numerical increase after the initial reduction highlights the need for longer-term evaluation of acute medication use after Kampo initiation.

An important finding of the present study was that MHD improved after Kampo initiation, whereas MMD did not show statistically significant changes. However, interpretation of this finding requires consideration of the clinical context. Before Kampo initiation, patients had already received ongoing CGRP-mAb therapy, and median MMD had decreased from 4.0 days before CGRP-mAb treatment to 1.0 day at baseline. Although 79.5% of patients were classified as having CM, CM classification was based on the diagnosis established at the initial clinical presentation. Baseline MMD values were assessed after ongoing preventive treatment, including CGRP-mAb therapy, and therefore reflected headache status at the time of Kampo initiation rather than at the time of CM diagnosis. Accordingly, the lack of further reduction in MMD after Kampo initiation may partly reflect a floor effect, as migraine frequency had already substantially improved before the introduction of Kampo therapy. In contrast, overall headache burden remained substantial, as reflected by persistent MHD, suggesting that the observed benefit of add-on Kampo therapy may have been more strongly related to residual non-migraine headache days or headache burden not fully controlled by CGRP-mAb treatment.

Work productivity impairment, assessed using the presenteeism score, also improved following Kampo therapy. Migraine is known to impose a substantial socioeconomic burden, primarily through reduced productivity rather than absenteeism [1,2,18,23,24]. Therefore, improvement in presenteeism may represent an important patient-centered benefit of add-on Kampo therapy. In contrast, the lack of significant improvement in absenteeism may partly reflect a floor effect because the median absenteeism score was already 0.0 at baseline, leaving little room for further improvement.

The findings of the present study are broadly consistent with previous reports suggesting a potential role for Kampo medicine in patients with insufficient response to conventional migraine therapies [12,14]. The present study extends these observations by evaluating a highly refractory migraine cohort receiving CGRP-mAb treatment. In this population, many patients had a history of multiple preventive medication use, prior CGRP-mAb switching, and re-worsening after an initial response. Previous reviews of treatment-resistant migraine have highlighted the limited therapeutic options available for patients who continue to experience substantial disability despite multiple preventive treatments [9]. In this setting, the favorable outcomes observed after Kampo initiation suggest that Kampo therapy may contribute to treatment optimization in a subset of highly refractory patients, even among patients who remain symptomatic despite modern CGRP-targeted therapies.

Oral CGRP receptor antagonists (gepants) have recently emerged as a novel treatment option for migraine, with growing real-world evidence supporting their effectiveness [25]. However, although gepants can be used for acute migraine treatment in patients receiving CGRP-mAbs, preventive co-administration of gepants and CGRP-mAbs is currently not available in Japan. Therefore, additional treatment optimization strategies remain necessary for patients who continue to experience substantial migraine burden during CGRP-mAb therapy. In this setting, Kampo therapy may represent a practical adjunctive option for selected patients who remain symptomatic despite CGRP-targeted treatment.

Other adjunctive approaches have also been proposed for patients with refractory migraine. Recent evidence suggests that lifestyle modifications, including optimization of sleep habits, physical activity, stress management, and dietary behaviors, may enhance treatment outcomes when combined with CGRP-targeted therapies [26]. In addition, dietary supplements and complementary therapies have been explored as potential adjunctive strategies for patients with persistent migraine burden despite conventional preventive treatment [27]. Therefore, Kampo therapy should be considered within the broader context of multimodal treatment optimization approaches rather than as a stand-alone intervention. Future studies are warranted to compare the relative and additive benefits of Kampo therapy, lifestyle interventions, and dietary supplements in highly refractory migraine populations.

The potential complementary mechanisms of Kampo and CGRP-targeted therapies may provide one possible explanation for the observed findings. Kampo formulations may influence pain-processing pathways that differ from CGRP pathway inhibition [10,11], potentially providing additional benefit in patients with persistent headache burden despite ongoing CGRP-mAb treatment. However, these mechanisms were not directly investigated in the present study and remain hypothetical.

Responder analysis showed that re-worsening after an initial response to CGRP-mAb therapy was more frequent among responders, whereas MOH status was more common among non-responders. Furthermore, re-worsening after an initial CGRP-mAb response showed an exploratory association with treatment response in the four-variable Firth penalized logistic regression model. The association remained evident in the reduced two-variable Firth model, supporting the consistency of the finding across model specifications. However, given the small sample size, the highly unbalanced distribution of re-worsening among responders, and the wide confidence intervals, this finding should be interpreted cautiously. Overall, 41.8% of treatment episodes achieved a ≥30% reduction in MHD at 1 month, which may be clinically meaningful given the highly refractory nature of the study population. One possible explanation is that patients with re-worsening after an initial response may retain responsiveness to preventive treatment despite a gradual loss of efficacy over time [7,28]. In such patients, persistent headache burden may result from mechanisms not fully addressed by CGRP pathway modulation alone, and additional symptom-targeted treatment with Kampo medicine may therefore provide incremental benefit.

Another important finding was the association between MOH status and reduced treatment responsiveness. MOH status was significantly more common among non-responders in the univariable analysis and was associated with a smaller percentage reduction in MHD in the sensitivity analysis. Excessive AMU is known to contribute to migraine chronification and may reflect greater disease severity and treatment complexity [9,29]. Therefore, MOH status may be associated with reduced responsiveness to adjunctive treatment strategies, including Kampo therapy. Taken together, these exploratory findings suggest that re-worsening after an initial CGRP-mAb response may be associated with response to add-on Kampo therapy, whereas MOH status may be associated with a smaller reduction in MHD.

Several limitations should be acknowledged. First, this was a retrospective single-center observational study, which may introduce selection bias and limit generalizability. Second, the sample size was relatively small, the number of treatment episodes decreased during follow-up, and follow-up was limited to 3 months. Therefore, the long-term durability of the observed effects and the possibility of later re-worsening after Kampo therapy could not be determined. Third, no control group was included; therefore, causal relationships between Kampo therapy and clinical improvement cannot be established. Fourth, because some patients contributed multiple treatment episodes, within-patient correlation was not explicitly accounted for in the statistical analyses. This potential non-independence may have affected statistical inference, and the episode-based results, particularly the regression findings, should therefore be interpreted as exploratory. Fifth, Kampo formulations were selected according to an institution-specific clinical algorithm, which may limit generalizability to other settings. Sixth, adverse events were assessed retrospectively from routine medical records; therefore, minor or unreported events may not have been fully captured. Seventh, socioeconomic variables such as educational attainment and income were not systematically collected and could not be evaluated. Finally, residual confounding may have influenced the observed outcomes. Given these limitations and the retrospective uncontrolled design, the present findings should be considered hypothesis-generating and require confirmation in prospective controlled studies.

## 5. Conclusions

Add-on Kampo therapy may represent a treatment optimization strategy for highly refractory migraine patients with inadequate response or re-worsening during CGRP-mAb therapy. Re-worsening after an initial CGRP-mAb response showed an exploratory association with treatment response, whereas MOH status was associated with a smaller percentage reduction in MHD in the sensitivity analysis. These hypothesis-generating findings require confirmation in prospective controlled studies.

## Figures and Tables

**Table 4:** Responder rates based on reduction in MHD after add-on Kampo therapy.

Outcome	Responders, *n*/N (%)	95% CI
≥30% reduction in MHD		
1 month	23/55 (41.8%)	[28.7%, 55.9%]
2 months	17/31 (54.8%)	[36.0%, 72.7%]
3 months	9/23 (39.1%)	[19.7%, 61.5%]
≥50% reduction in MHD		
1 month	7/55 (12.7%)	[5.3%, 24.5%]
2 months	7/31 (22.6%)	[9.6%, 41.1%]
3 months	2/23 (8.7%)	[1.1%, 28.0%]

Responder rates at each time point were calculated using available-case analyses. MHD: monthly headache days, CI: confidence interval.

**Table 5:** Factors associated with treatment response at 1 month.

Variable	Responder (*n* = 23)	Non-Responder (*n* = 32)	*p* Value
Age, years	48.0 (36.5, 51.0)	44.0 (39.0, 52.3)	0.492
Chronic migraine, *n* (%)	17 (73.9)	30 (93.8)	0.057
Medication-overuse headache, *n* (%)	10 (43.5)	25 (78.1)	0.012
CGRP-mAb switch history, *n* (%)	13 (56.5)	18 (56.3)	1.000
Re-worsening after an initial response, *n* (%)	22 (95.7)	18 (56.3)	0.002
Time from CGRP-mAb initiation to Kampo initiation, months	11.5 (8.5, 15.8)	8.0 (4.0, 16.0)	0.935

Data are presented as median (interquartile range [IQR]) or *n* (%). Responder was defined as ≥30% reduction in MHD at 1 month. *p* values were calculated using the Mann–Whitney U test or Fisher’s exact test, as appropriate. CGRP-mAb: calcitonin gene-related peptide monoclonal antibody.

**Table 6:** Firth penalized multivariable logistic regression analysis of factors associated with treatment response at 1 month.

Variable	Penalized OR	95% CI	*p* Value
Medication-overuse headache	0.399	[0.11, 1.38]	0.147
CGRP-mAb switch history	0.463	[0.08, 2.21]	0.341
Re-worsening after an initial response	14.581	[2.15, 180.80]	0.005
Time from CGRP-mAb initiation to Kampo initiation	0.977	[0.90, 1.05]	0.534

The dependent variable was responder status, defined as a reduction of 30% or greater in monthly headache days at 1 month after Kampo initiation. The model included medication-overuse headache, CGRP-mAb switch history, re-worsening after an initial response, and time from CGRP-mAb initiation to Kampo initiation. ORs, 95% CIs, and *p* values were estimated using Firth penalized logistic regression. CGRP-mAb: calcitonin gene-related peptide monoclonal antibody; CI: confidence interval; OR: odds ratio.

**Table 7:** Multiple linear regression analysis of factors associated with the percentage reduction in monthly headache days at 1 month after Kampo initiation.

Variable	β Coefficient	95% CI	*p* Value
Medication-overuse headache	−0.123	[−0.241, −0.004]	0.042
CGRP-mAb switch history	−0.025	[−0.161, 0.111]	0.717
Re-worsening after an initial response	0.190	[0.039, 0.341]	0.015
Time from CGRP-mAb initiation to Kampo initiation	−0.003	[−0.010, 0.004]	0.385

The dependent variable was the percentage reduction in monthly headache days (MHD) at 1 month after Kampo initiation. Positive β coefficients indicate a greater percentage reduction in MHD, whereas negative β coefficients indicate a smaller percentage reduction. Variables were included in the model based on clinical relevance and sample size considerations. CGRP-mAb: calcitonin gene-related peptide monoclonal antibody; CI: confidence interval; MHD: monthly headache days.

## Data Availability

The datasets used and analyzed during the current study are available from the corresponding author on reasonable request.

## Supplementary Materials

The following supporting information can be downloaded at: https://www.mdpi.com/article/10.3390/jcm15218633/s1. Supplementary Table S1. Reduced Firth penalized logistic regression analysis of factors associated with treatment response at 1 month after Kampo initiation.
